# Bacteriological and Physicochemical Studies on Tigris River Near the Water Purification Stations within Baghdad Province

**DOI:** 10.1155/2012/695253

**Published:** 2012-12-24

**Authors:** Khalid K. Al-Bayatti, Kadhum H. Al-Arajy, Seba Hussain Al-Nuaemy

**Affiliations:** ^1^College of Pharmacy, University of Al-Mustansiriya, P.O. Box 14070, Baghdad, Iraq; ^2^College of Science, University of Al-Mustansiriya, P.O. Box 14070, Baghdad, Iraq

## Abstract

We studied the physical, chemical, and microbiological factors that influence drinking water quality processed from River Tigris, and of the three main drinking water purification stations located at different parts of Tigris River, along with evaluation of drinking water of Al-Shula region in Baghdad city. Water samples were taken monthly from December 2009 to September 2010. Physical and chemical analyses of water included determination of temperature, pH, turbidity, electrical conductivity, total dissolved solids, salinity, dissolved oxygen, and biological oxygen demand. The results of water before and after purification indicated values within the international allowable levels. Microbial analyses included estimation of the number of total viable microbial counts, total coliform, total fecal *E. coli* and *Pseudomonas aeruginosa*, and other pathogenic bacteria that might be present in the water of the three stations and of the Tigris River, and also the tap water from Al-Shula houses. The results indicated that the types and proportions of various bacterial species isolated from different water sources were almost similar. This indicates inefficient purification procedures in all the stations studied, which exceeded the internationally allowable level of pathogens in potable water. Also, this explains the high incidence rate of children diarrheal reported in Al-Shula region.

## 1. Introduction

Safety and quality of drinking water are always an important public health concern [1, 2]. The raw water quality can be affected by human or animal activity either within that body of water or within its watershed. 

According to UNICEF report, about 800 million people in Asia and Africa are living without access to safe drinking water. Consequently this has caused many people to suffer from various diseases [3]. However, inadequate quantity, poor quality of drinking water, and poor sanitation are the main reasons in incidence and prevalence of diseases in the world [4].

Contamination of water has been frequently found associated with transmission of diseases causing bacteria, *Vibrio*, *Salmonella*, bacterial and parasitic dysentery, and acute infection diarrhea causing *E. coli *[5]. Poor sanitation and food sources are integral to enteric pathogen exposure. Drinking water is a major source of microbial pathogen and considered to be one of the main reasons for increased mortality rates among children in developing countries [6].

 Comprehensive evaluations of microbial quality of water require survey of all the pathogens that have potential for human infections [7]. World Health Organization (WHO) essential parameters of drinking water quality are fecal *Escherichia coli *and total coliforms, chlorine residual, turbidity, pH, dissolved oxygen content, and temperature [8]. These guidelines are essential determinants to reduce or eliminate the risk of water pollution. While the drinking water resources, contaminated with agricultural, industrial, and sewage waste are dangerous and less usable for human consumption and for agricultural purposes.

 Water Quality Index (a single value indicator of the water quality) was analysed by Alobaidy et al. [9] to evaluate the raw and treated drinking water from Tigris River within Baghdad. Using this approach Alobaidy and his colleagues showed that Tigris water never reached “Excellent” level nor fallen to “unsuitable” condition, except in occasional untreated water sample. It is thus important to study and know the physical, chemical, and biological nature of this water to ascertain hygienic quality of water sources for human consumption and for general community purposes. WHO drinking water quality guidelines and Iraqi standards both recommend that fecal coliform must not exist in 100 mL of water sample [4, 10].

In Baghdad city there are seven water treatment stations located on the banks of the Tigris River along a distance of 50–60 km. These stations are Sharq Diglla station, Al-Karama station, Al-Wathba station, Al-Qadisia station, Al-Dorra station, Al-Wahda station, and Al-Rashed station. These stations are providing Baghdad with most of its water requirements.

In recent years several M.Sc. and Ph.D. theses have been conducted in Iraq concerning drinking water quality and the impacts of some of the bacteriological and ecological factors on the Tigris River [11–14].

The aim of this work is to study different parameters of drinking water that affect the health of people of Baghdad city. To perform this target, four factors were studied first, the microbiologic survey of different pathogens of water of Tigris River and of three water purification stations, Sharq Diglla, Al-Karama, and Al-Qadisia. Second aim was to study the physical and chemical factors that influence water quality of the Tigris River. Third aim was to evaluate the treatment efficiency of the 3 stations. The last aim was to evaluate drinking water of Al-Shula region in Baghdad city that is provided with water from Al-Karama purification station. 

## 2. Materials and Methods

### 2.1. Study Locations

Baghdad has an area of 800 km^2^, and 65% of all the industrial institutions and factories were located in Baghdad. This condition generated ecological problems threatening the ecosystem of Baghdad city, due to the drainage of sewages and byproducts of these institutions and factories directly to the body of Tigris River [15].

The three purification stations were chosen according to their purification capacity and locations along the Tigris River. They provide Baghdad city with 910000 m^3^/day and comprise about 72% of all the 7 purification stations capacity present in Baghdad. 

#### 2.1.1. Sharq Diglla Station

**Figure 1:**
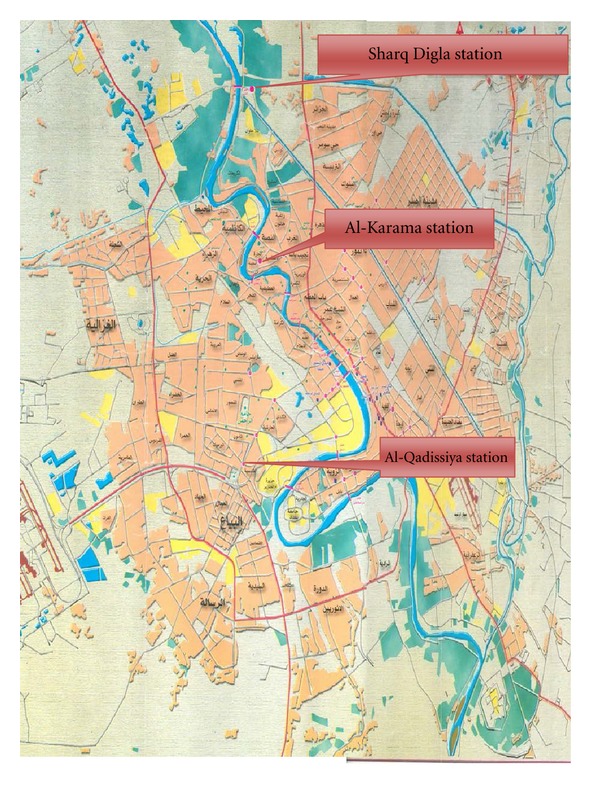
The locations of the three purification stations in Baghdad province.

The station is located at the North of Baghdad and its purification capacity is about 650000 m^3^/day, it provides Al-Rusafa side of Baghdad with drinking water, and it comprises about 51% of the purification capacity of all the stations in Baghdad (Figure 1).

#### 2.1.2. Al-Karama Station

The station is located at the center of Baghdad and its purification capacity is about 160000 m^3^/day, it provides part of Al-Rusafa and part of Al-Karkh sides of Baghdad with drinking water, and it comprises about 13% of the purification capacity of all the stations in Baghdad (Figure 1). 

#### 2.1.3. Al-Qadisia Station

The station is located at the south of Baghdad and its purification capacity is about 100000 m^3^/day, and provides Al-Qadisia region with drinking water and it comprises about 8% of the purification capacity of all the stations in Baghdad (Figure 1).

### 2.2. Sample Collection

Duplicate sample of raw water from Tigris River and from stations after purification were taken every month from December 2009 to September 2010. Forty tap water samples were also taken from the houses of Al-Shula region during summer months, June, July, August, and September 2010.

### 2.3. Bacteriological Tests

Raw water samples were collected in clean sterilized glass bottles of 250 mL capacity from 20 to 30 cm under the surface of water of Tigris River from the point where it inters to the pipe of purification station. Drinking water samples from the station were also taken in sterilized bottles of 250 mL capacity. Forty 200 mL tap water sample were also taken from the houses of Al-Shula region after the sterilization of house faucet. All these bottles were closed carefully and transported to the laboratory on ice, and kept at 4°C, and processed within 6 hrs. Microbiological survey of water samples including total viable bacterial count were performed according to the general standard methods for examination of water and waste water [16]. Total coliform, fecal *E. coli*,  and *Pseudomonas aeruginosa* was determined by means of standard coliform fermentation technique including presumptive, confirmed, and completed tests [17]. For identification of other pathogenic enteric bacteria different dilutions of water samples from different sources were spread on Nutrient agar, Macconkey agar, blood agar, eosin-methylene blue agar (EMB), and Thiosulfate citrate bile sucrose agar (TCBS) medium. The plates were incubated overnight at 37°C, and after incubation, cultures were examined for distinct colonies. These colonies were streaked on nutrient agar slant and incubated for 24 hrs at 30°C, and kept as stock cultures. Conventional bacteriological methods [18] and API_20E_ were used for identification of each isolate.

### 2.4. Physical and Chemical Tests

Glass containers of 1 L capacity were used to collect raw water samples and drinking water from the stations faucets as described for bacteriological analyses, except for dissolved oxygen (DO) and biological oxygen demand (BOD) tests, dark glass container of 300 mL capacity were used. These containers were transported to the laboratory on ice and kept at 4°C and analyzed within 24 hrs. Chemical and physical measurements of the water samples were done as follows.

#### 2.4.1. Temperature

The temperature of the raw water of the Tigris River was measured by suspending a thermometer about 10 cm below the surface of water for at least 2 minutes before taking the reading, while the temperature of the stations water after purification were taken by putting the thermometer under the running water from the faucet for 5 minutes before taking the temperature [16].

#### 2.4.2. Hydrogen Ion Concentration pH

The pH-meter (SCHOTT) was used. 

#### 2.4.3. Turbidity

Turbidity meter (SCHOTT) was used, the turbidity is expressed in Nephelometric Turbidity unit (NTU). 

#### 2.4.4. Electrical Conductivity

The conductivity meter (SCHOTT) was used, and the EC was determined according to the method described by Golterman et al. [19] and it is expressed in microsiemens/cm. 

#### 2.4.5. Total Dissolved Solid Material (TDS)

The Kondukto meter (SCHOTT) was used and the TDS is expressed in mg/L. 

#### 2.4.6. Dissolved Oxygen (D.O)

The (YSI model 54A) oxygen-meter was used to determine the D.O, and is expressed in mg/L. 

#### 2.4.7. Biological Oxygen Demand (BOD)

The first reading of D.O was taken with oxygen meter and after 5 days of incubation of water samples in 20°C without light, the second reading of D.O was taken. The BOD was determined by the below equation and expressed in mg/L

(1) $$\begin{array}{c} \text{BOD}=\left(\text{D}.\text{O}1-\text{D}.\text{O}2\right)\;\;\text{mg}/\text{L}. \end{array}$$

#### 2.4.8. Salinity

The conductivity value was determined according to the method described in [19]. 

### 2.5. Statistical Analysis

Statistical approaches, like LSD, ANOVA, Chi-square test, Kruskal-Wallis test, and correlation test for all the factors during the studied period and for the 3 stations, were done by analyzing the data (*P* < 0.05) using SPSS Ver. (10.0). 

### 2.6. Results and Discussion

In order to assess the water quality of a particular pond, stream or lake, we conducted tests to determine the level of dissolved oxygen, biological oxygen demand, nitrate, and phosphate in the water as well as the pH, temperature, and turbidity. These parameters along with the microbial contamination of water like total coliform and fecal *E. coli* were considered good indicators of the quality of water [20].

 Common approach in determining amount of microbial safety of public water distribution system is based on sampling strategies in consumption point or water faucet [21]. Therefore sampling method is a critical factor in determining the microbial quality of distribution system, since according to the drinking water standards [4], the number of coliform and fecal coliform must be zero in any situation, thus raw water supply and tap water were selected as sampling point and all measurements were done according to [16].

Table 1 shows the confidence limit for GLM of all variables of physical, chemical, and microbial isolates studied with respect to seasons, months, stations, and replicate samples. The results are briefly described.

#### 2.6.1. Temperature

There was a high significant difference with respect to seasons and months, air, and raw water temperature, *P* < 0.01, whereas no significant difference was recorded with respect to stations temperature. The highest regression value for air temperature was 0.98 and was 0.99 for both Tigris raw water and stations water.

#### 2.6.2. pH

No significant difference were recorded between seasons, months, and sites, *P* ≥ 0.05 and regression (*R*) values were 0.22 for stations and 0.42 for raw water.

#### 2.6.3. Turbidity

Data revealed turbidity values less than 5 NTU of drinking water in the stations. The turbidity of supply water is mostly used as a measure of water quality in water treatment plants. The desirable level less or equal to 1 NUT was recommended by WHO. Turbidity value up to 5 NUT will indicate inadequate efficiency of treatment plant and possibly correlate with increased total coliform bacteria [22]. The maximum Iranian standard of turbidity for drinking water is 55 NTU [23]. No significant difference for turbidity was seen with respect to seasons and months *P* ≥ 0.05, whereas highly significant difference was recorded for sites *P* < 0.01 and the *R* for the 3 stations was 0.41. The turbidity of the raw water of Tigris was highly significant with respect to the seasons and sites *P* < 0.01, and no significant difference with respect to month *P* ≥ 0.05 and the *R* value was 0.05.

#### 2.6.4. Electrical Conductivity

Electrical conductivity in the aquatic ecosystem is considered to be a good indicator for evaluating total dissolved solid materials in water and nature of the purity of water [16]. The EC of the 3 stations ranges between 655–734 microsiemens/cm and this range is within the Iraqi acceptable limit (2000 microsiemens) for drinking water [24]. The data show high significance *P* < 0.01 with respect to the seasons, months and stations and the *R* value was 0.77 and for raw water was 0.61.

#### 2.6.5. Salinity and Total Dissolved Solid

Salinity and TDS have high significant difference with respect to seasons, months, and stations. The *P* < 0.01 and the regression values were 0.74 and 0.75 for drinking water and 0.57 and 0.67 for raw water for both TDS and salinity, respectively.

#### 2.6.6. Dissolved Oxygen (D.O) and Biological Oxygen Demand (BOD)

D.O and BOD also have high significant difference with respect to seasons, months, and stations. The *P* < 0.01 and the regression values for D.O and BOD were 0.79 and 0.4 respectively.

### 2.7. Bacteriological Analysis

Bacteriological analysis of water resources included total viable bacterial counts, total coliforms, total *E. coli,* and total *Pseudomonas aeruginosa*. Data in Table 2 indicate the presence of at least 14 species of bacteria belonging to the family Enterobacteriaceae and some other species belong to the family Pseudomonadaceae. The most common species were *E. coli* and *Pseudomonas aeruginosa.* The presence of these 2 species indicate that the drinking water are most probably contaminated with human and animal feces [17]. The data indicated a high level of total viable bacterial counts in water of the 3 stations and were between (1–64 CFU/100 mL). These microbial findings indicate a non-efficient purification procedures in all stations studied. Also, these microbial counts were exceeding the international allowable levels especially in the Al-Karama station, while the total viable bacterial counts of the Tigris river (raw water) was between (468–9100 CFU/mL). These numbers were indicated a high significant differences for seasons, months, and sites for the three stations (drinking water) and also for raw water. The *P* < 0.01 and regression values were 0.59 and 0.79 for drinking and raw water, respectively. 

 For total coliform and fecal *E. coli*, the lowest and highest average number for the 3 stations water were 1.1 and 7.1 CFU/100 mL, respectively; again these numbers exceeded the international allowable consumption level for drinking water [17]. A highest average numbers were recorded in spring and summer seasons 3.3 and 3.7 CFU/100 mL for total coliform and 2.1 and 1.5 CFU/100 mL for fecal *E. coli*. While the lowest average numbers were recorded during winter and autumn seasons 0.0 and 0.7 CFU/100 mL for total coliform and 0.0 and 0.3 CFU/100 mL for fecal *E. coli*. These numbers indicated that there are high significant difference for total coliform and fecal *E. coli* in drinking water with respect to seasons and months *P* < 0.01, and no significant difference was seen with respect to the sites (stations), *P* > 0.05. The regression values were 0.71 and 0.59 for total coliforms and fecal *E. coli*, respectively.

 In Tigris River water, on the other hand, the total coliform counts exceeded (1795–63000 CFU/100 mL) while the pathogenic fecal *E. coli* counts were between (335–39000 CFU/100 mL). These indicated a high significant difference for total coliform and fecal *E. coli* in raw water with respect to seasons and sites (stations) *P* < 0.01, and significant difference was seen with respect to months *P* < 0.05. The regression values were 0.57 for total coliform and 0.55 for fecal *E. coli*.

 The percentages of total coliform with respect to total viable bacterial count were 20.4% and 32% for cold and warm seasons, respectively while the percentages of fecal *E. coli* were 22.4% and 31.7% for cold and warm seasons, respectively. On the other hand the *Pseudomonas aeruginosa* counts ranged from 2 to 21 CFU/100 mL in the 3 stations and the highest level recorded was in summer season. These numbers indicate high significant difference with respect to seasons and months *P* < 0.01 and no significant difference with respect to stations *P* > 0.05. The regression values were 0.59 for the 3 stations whereas the number of *Pseudomonas aeruginosa* was between 580 and 160000 CFU/100 mL in Tigris River. These indicate a high significant difference with respect to seasons and stations *P* < 0.01 and no significant difference with respect to months *P* ≥ 0.05. The regression value was 0.46 for the *Pseudomonas aeruginosa* in raw water (Table 1). Other bacterial species were also isolated from the water resources namely *Aeromonas hydrophila, Vibrio cholera,* and *Vibrio fluvialis.*

 On the other hand, the number and different bacterial species isolated from different water resources were also investigated (Table 2). The dominant bacterial species were also *E. coli, Klebsiella pneumonia,* and *Pseudomonas aeruginosa*. The data in (Table 2) showed high coincidence between the types and percentages of the main bacterial species isolated from different water sources and from patients children with diarrhea in Al-Shula region,* E. coli *27%, *Klebsiella pneumonia *30%, *Pseudomonas aeruginosa *23%, *Citrobacter freundii *14%, *Pseudomonas fluorescens* 3%, and *Serratia plymuthica *3% [25].  The species *Klebsiella pneumonia* was significantly different, while* E. coli* and *Pseudomonas aeruginosa* were not significantly different in (M-Z) test between the three sources of water (Table 2). These results coincident with the result of other researchers that the diarrheal cases is almost resulted from consumption of contaminated drinking water with various types of microbial agent especially *E. coli *[26].

The highest numbers of total coliform and fecal *E. coli* recorded for tap water in houses of Al-Shula region were 16 CFU/100 mL and 9.2 CFU/100 mL, respectively, during august 2010 while the lowest numbers were 1.1 CFU/100 mL and 0.0 CFU/100 mL during July 2010 respectively (Table 3). These numbers indicate a significant difference during August 2010, *P* < 0.05 (Table 4). Therefore we conclude that the high contamination of drinking water in Al-Shula houses with different diarrhea causing bacteria like coliform, and fecal *E. coli* and others. This indicate that people from these areas might prone to water-borne diseases, and these all might reside behind the large number of diarrheal cases that have been seen in patients attending Al-Hakeem hospital at Al-Shula region.

 It is noteworthy, however, that some fecal pathogens including many viruses and protozoa, may be more resistant to treatment by chlorine than the indicator bacteria [27], this implies that even a low level of contamination measured by bacteriological analysis may be a risk, especially during an outbreak of diarrhea like cholera [10].

According to the bacteriological data investigated in this research, it was possible to analyze the efficiency of the 3 stations according to the average total number of viable microbial agents in (raw water) and out (drinking water) of the purification units of the three stations using the following equation.

Efficiency = output/input × 100,
efficiency of Sharq Diglla station = 0.55 and it comprises 49%,
efficiency of Al-Karama station = 0.16 and it comprises 14%,
efficiency of Al-Qadisia station = 0.41 and it comprises 37%.

**Figure 2:**
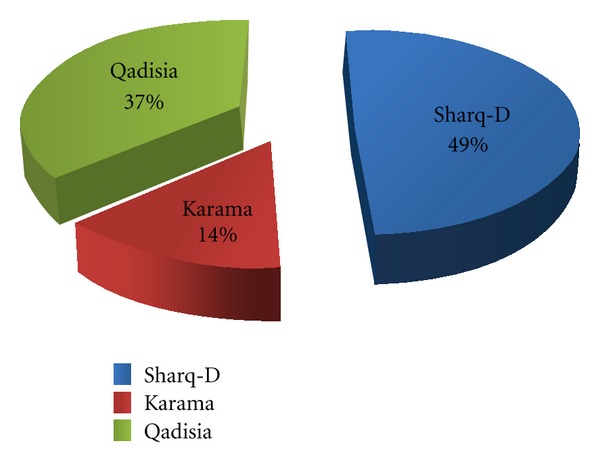
The percentage of the efficiency of the 3 purification stations in Baghdad.

Accordingly, the efficiency of sharq Diglla station in purification process is better than both stations followed by Al-Qadisia station, then Al-Karama station Figure 2.

For raw water, the water quality showed high concentration of bacteria upstream to downstream of Tigris River. Physicochemical parameters, on the other hand, were within the acceptable values. This might be due to pollution of raw water from urban wastes and other sources. Treated water quality showed to be suitable but not good for public consumption in Sharq Diglla station (55%) efficiency and in Al-Qadisia station (41%) efficiency while it showed unacceptable water quality (14%) efficiency in Al-Karama station. These results were in accordance with the results obtained by Alobaidy et al. [9]. They concluded that the raw water of Tigris is poorest in quality throughout the year as the efficiency of Water Treatment Plants ranges from 25.07 to 63.30 in a whole period of 7 years of study. Therefore, the Authority should strive to control various procedures employed for treating and purification process in all the purification stations in Baghdad city. Ultimately, reconsideration of the water treatment stations system is needed since these stations were designed to provide physical and biological treatment rather than chemical treatment of raw water [13].

## 3. Conclusions

According to the results obtained, we conclude with the following.

1. Total coliform counts and fecal *E. coli *counts at the three stations in all seasons were more than the international permissible levels recommended by WHO. The level at Al-Qadisia station was much less than the level at Al-Karama station. This might due to heavy contamination of Tigris River with waste water discharged from more than 15 untreated Al-Rusafa and Al-Karkh sewage upstream of Al-Karama station intake. Therefore, strict measures should be taken in order to control the levels of pollutants discharged into Tigris River.
2. Water quality in all water treatment stations with respect to temperature, pH, turbidity, salinity, and E.C are all within the water quality standards recommended by Iraqi and WHO standards except TDS. Total dissolved solids was increased in treated water than in river water. This could be due to the addition of Alum to the water during the coagulation process and to the absence of any chemical treatment unit in Baghdad water treatment process.
3. The efficiency of Sharq-Diglla station in purification process is better than both Al-Qadisia and Al-Karama purification stations.
4. Drinking water in Al-Shula region was not suitable for human consumption due to high contamination levels with Total coliform and Fecal *E. coli*. Therefore, we recommend further studies in other regions of Baghdad city with respect to suitability of drinking water for human consumption provided by different water purification stations.
5. The Authority of Baghdad Mayoralty should practice more efforts in controlling various procedures employed for treating and purification process in all the purification stations.

## Figures and Tables

**Table 1:** Confidence limit of GLM of all the variables studied with respect to seasons, months, stations, and replicate samples.

Variables studied	SOV						*R* Squared
	Corrected Model	Intercept	Season	Month	Station	REP
Salinity (mg/L) of water of 3 stations	0.00	0.00	0.00	0.00	0.01		0.74
TDS (mg/L) of water of 3 stations	0.00	0.00	0.00	0.00	0.00	0.08	0.74
EC (microsemens) of water of 3 stations	0.00	0.00	0.00	0.00	0.01	0.53	0.77
Turbidity NTU of water of 3 stations	0.00	0.00	0.00	0.00	0.00	0.43	0.41
pH of water of 3 stations	0.01	0.00	0.15	0.34	0.00	0.53	0.22
No. of *Pseudomonas aeruginosa*/100 mL of water of 3 stations	0.41	0.00	0.43	0.59	0.08	0.85	0.59
No. of *E*. *coli*/100 mL of water of 3 stations	0.00	0.00	0.00	0.01	0.96	0.57	0.59
Total coliform/100 mL of water of 3 stations	0.00	0.00	0.00	0.01	0.96	0.57	0.71
Total number of bacteria of water of 3 stations	0.00	0.00	0.00	0.00	0.22	0.26	0.59
Temperature °C of water of 3 stations	0.00	0.00	0.00	0.00	0.01	0.81	0.99
Temperature °C of air	0.00	0.00	0.00	0.00	0.16		0.98
Temperature °C of water of Tigris River	0.00	0.00	0.00	0.00	0.34		0.99
BOD mg/L of water of Tigris River	0.01	0.00	0.03	0.04	0.05	0.97	0.40
DO mg/L of water of Tigris River	0.00	0.00	0.00	0.00	0.01	0.91	0.79
Salinity (mg/L) of water of Tigris River	0.00	0.00	0.00	0.00	0.00	0.69	0.67
TDS (mg/L) of water of Tigris River	0.00	0.00	0.00	0.00	0.21	0.57	0.57
EC (microsiemens) of water of Tigris River	0.00	0.00	0.00	0.00	0.00	0.52	0.61
turbidity NTU of water of Tigris River	0.00	0.00	0.00	0.70	0.00	0.47	0.50
pH of water of Tigris River	0.01	0.00	0.01	0.02	0.18	0.30	0.42
No. of *Pseudomonas aeruginosa*/100 mL of water of Tigris River	0.00	0.00	0.01	0.06	0.01	0.30	0.46
No. of *E*. *coli*/100 mL of water of Tigris River	0.00	0.00	0.00	0.02	0.00	0.78	0.55
Total no. of coliform/100 mL of water of Tigris River	0.00	0.00	0.00	0.05	0.00	0.97	0.57
Total no. of bacteria/100 mL of water of Tigris River	0.00	0.00	0.00	0.01	0.00	0.94	0.78

**Table 2:** Number and types of bacterial species isolated from different water sources.

Bacterial species	Number and types of bacterial species isolated from			M-Z test*	C.S.
	Drinking water of Al-shula houses	Water of stations	Water of Tigris River
*E*. *coli *	8	10	30	0.885	NS
*Serratia plymuthica *	1	0	0	4.995	NS
*Klebsiella ornithiolytica *	0	0	1	0.749	NS
*Klebsiella pneumonia *	9	4	14	6.856	S
*Klebsiella terrigena *	0	0	0	—	—
*Pseudomonas aeruginosa *	7	16	30	1.169	NS
*Pseudomonas fluorescens *	1	1	2	0.207	NS
*Citrobacter freundii *	4	2	5	3.243	NS
*Pseudomonas paucimobilis *	0	1	1	0.824	NS
*Sphingomonas paucimobilis *	0	2	1	2.815	NS
*Pantoea *spp.	0	2	2	1.666	NS
*Rahnella aquatilis *	0	1	2	0.625	NS
*Enterobacter sakazakii *	0	3	1	5.309	NS
*Enterobacter cloacae *	0	1	4	1.378	NS
*Enterobacter aerugena *	0	0	0	—	—
*Proteus mirabilis *	0	0	1	0.742	NS
*Aeromonas hydrophila *	0	1	2	0.625	NS
*Vibrio cholera *	0	0	3	2.251	NS
*Vibrio fluvials *	0	0	3	2.251	NS
*Serratia ficaria *	0	1	1	0.824	NS
*Salmonella orizonae *	0	1	0	2.909	NS

Total	30	46	103	8.851**	S

*Chi-Square test with 2 d.f. (*α* = 0.05) = 5.991 & (*α* = 0.01) = 9.210.

**Kruskal Wallis test recorded (Sig. at *P* = 0.012, i.e., Sig. at *P* < 0.05).

**Table 3:** Statistical analyses of the total coliform and fecal *E*. *coli* from tap water of Al-Shula houses.

95% confidence		Standard deviation	Mean	Sample	Months	Indicators
Higher limit	Lower limit					
0.36	0.00	0.35	0.11	10	June	Total coliform
1.11	0.00	0.78	0.55	10	July	
7.49	0.00	5.31	3.69	10	August	
1.85	0.15	1.19	1.00	10	September	
2.3	0.38	3.00	1.34	40	Total	

0.00	0.00	0.00	0.00	10	June	Fecal* E*. *coli *
0.72	0.00	0.70	0.22	10	July	
4.79	0.00	3.52	2.27	10	August	
1.68	0.00	1.26	0.78	10	September	
1.47	0.17	2.03	0.82	40	Total	

**Table 4:** Significant comparison (least sig. differences) of total coliform and fecal *E*. *coli* (CFU/100 mL) in drinking water of houses of Al-Shula.

September	August	July	Months	Indicators
0.475	0.006	0.723	June	Total coliform
0.717	0.015		July	
0.036			August	

0.364	0.011	0.797	June	Fecal *E*. *coli *
0.514	0.021		July	
0.088			August	
